# Tambulin Targets Histone Deacetylase 1 Inhibiting Cell Growth and Inducing Apoptosis in Human Lung Squamous Cell Carcinoma

**DOI:** 10.3389/fphar.2020.01188

**Published:** 2020-08-12

**Authors:** Wuming Wang, Yuzhen Liu, Long Zhao

**Affiliations:** Department of Thoracic Surgery, Jiangxi Provincial Chest Hospital, Nanchang, China

**Keywords:** tambulin, lung squamous cell carcinoma, apoptosis, histone deacetylase 1, B-cell lymphoma 2, caspase

## Abstract

There is an urgent unmet need to develop new therapeutics for lung squamous cell carcinoma (LSCC) as the current gold standard treatment regimens are dominated by chemotherapy. In this study, we observed the treatment effects of the natural compound tambulin on LSCC and explored its mechanism of action. LSCC cell lines H226 and H520 were cultured *in vitro* to observe the effects of tambulin on cell proliferation and apoptosis. Western blotting was used to detect the expression of histone deacetylase 1 (HDAC1) and apoptosis-related proteins. Cell derived xenografts (CDX) of H226 and H520 in nude mice were established to examine the inhibitory effects of tambulin *in vivo*. Results showed that tambulin inhibited the proliferation of H226 and H520 cells in a dose-dependent manner and inhibited the growth of CDX tumors. Tambulin also promoted the apoptosis of H226 and H520 cells, up-regulated the protein expression of cleaved caspase-3, cleaved caspase-9 and Bax, and down-regulated HDAC1 and Bcl-2 protein expression. In support of this, immunohistochemical analysis of CDX tumors from mice treated with tambulin showed increased expression of cleaved caspase-3 and Bax, while the expression of HDAC1 and Bcl-2 were decreased. What’s more, when HDAC1 was over-expressed *via* adenovirus transduction in H226 or H520 cells, the effects of tambulin were significantly attenuated. Interestingly, we found that combining tambulin with cisplatin treatment in CDX models was more effective than single drug treatment, suggesting that tambulin may enhance the sensitivity of LSCC to cisplatin. Taken together, this study proves that tambulin has a definite therapeutic effect on LSCC. Mechanistically, tambulin downregulates HDAC1, which in turn regulates the Bcl-2/caspase signaling pathway and promotes cancer cell apoptosis.

## Introduction

Lung carcinoma is a serious threat to human health and the number of annual deaths from lung carcinoma is higher than that of any other cancer (Ugo et al., 2018). In China, lung carcinoma is the most common cancer in men and the second most common cancer in women, while it has the highest mortality rate of all cancers regardless of gender (Zheng et al., 2019). According to the histological classification, lung carcinoma is divided into small cell lung carcinoma (SCLC) and non-small cell lung carcinoma (NSCLC). NSCLC accounts for 85%‑90% of all lung carcinomas, with lung squamous cell carcinoma (LSCC) being the second most common type of NSCLC after adenocarcinoma (Herbst et al., 2008). However, unlike the increasingly improved situation of targeted treatments for lung adenocarcinoma, there is currently no effective adjuvant treatment for LSCC in addition to ordinary chemotherapy (Scagliotti et al., 2008; Perez-Moreno et al., 2012). Therefore, actively exploring new therapeutics to combat LSCC is clinically significant.

Histone deacetylase 1 (HDAC1) is an important epigenetic protein, which can antagonize the acetylation of histones and non-histone proteins. HDAC1 is over-expressed in many types of tumors and is closely related to the clinical characteristics and prognosis of patients (Zhang et al., 2005; Fritzsche et al., 2008; Adams et al., 2010). Study shows that HDAC1 is over-expressed in lung carcinoma and that it is more highly expressed in LSCC than in lung adenocarcinoma (Cao et al., 2017). Compared to patients with high expression of HDAC1, patients with low expression have a better overall survival (Cao et al., 2017). Knock-down of HDAC1 inhibits the invasion of NSCLC and induces apoptosis (Zhang et al., 2018). This indicates that HDAC1 is a potential target for NSCLC treatment.

Natural compounds are highly sought-after medicinal interventions. Tambulin (**Supplementary Figure 1**) is a hydroxy-substituted flavanol extracted from *Zanthoxylum armatum* (DC) fruit. *Zanthoxylum armatum* is a Rutaceae plant which is used as cooking spice in China, India, and Nepal. Tambulin has been found to have a variety of biological activities, including vasodilation (Chen et al., 1999), anti-diabetic effects (Hameed et al., 2019), anti-oxidative effects (Pandey et al., 2019), and anti-cancer effects (Nooreen et al., 2017). In preliminary experiments, we observed that tambulin significantly inhibited the proliferation of human LSCC cell lines H226 and H520. Moreover, tambulin intervention significantly down regulated the protein expression of HDAC1. Therefore, the purpose of present study was to further verify the anti-LSCC effect of tambulin and to explore the relationship between its mechanism and HDAC1.

## Materials and Methods

### Reagents

Tambulin (purity > 98%) was obtained from the National Institute for the Control of Pharmaceutical and Biological Products (Beijing, CHN) and dissolved in dimethylsulfoxide (DMSO) (20 mg/ml). Cisplatin injection was purchased from Hansoh Pharma (Lianyungang, Jiangsu, CHN). HDAC1 rabbit polyclonal antibody (ab19845), cleaved caspase-3 rabbit polyclonal antibody (ab2302), cleaved caspase-9 rabbit polyclonal antibody (ab2324), B-cell lymphoma 2 (Bcl-2) rabbit monoclonal antibody (ab32124), and Bcl-2-associated X (Bax) rabbit monoclonal antibody (ab32503) were acquired commercially from the Abcam (Cambridge, Cambs, United Kingdom). The Annexin V-FITC apoptosis detection kit was obtained from eBioscience (San Diego, CA, United States).

### Cell Culture

The human normal lung epithelial cells BEAS-2, and human LSCC cell lines NCI-H226 and NCI-H520 were obtained from Procell (Wuhan, Hubei, CHN). BEAS-2 cells were grown in BEAS-2B cell specific medium (Procell, CHN). H226 and H520 cells were grown in Roswell Park Memorial Institute (RPMI) 1640 with 10% calf bovine serum and 1% penicillin-streptomycin at 37°C with 5% CO_2_ (v/v). A 293T cell line (Type Culture Collection of the Chinese Academy of Sciences, Shanghai, CHN) was grown in Dulbecco’s modified Eagle’s medium (DMEM) with calf bovine serum (10%) and penicillin-streptomycin (1%) at 37°C with 5% CO_2_ (v/v). Medium was replaced 2 to 3 days and the cells were passaged when the cell adherence area reached 80% of the culture dish.

### Construction of Recombinant Adenovirus

Site-specific recombination cloning was used to clone HDAC1 (GeneID: 3065) into GV287 vector (Shanghai Genechem Co., Ltd., Shanghai, China). Plasmids containing HDAC1 were transfected into 293T cells using envelope and packaging plasmids. Harvested virus from the supernatant by density gradient centrifugation and stored at -80°C. Virus titer was calculated using the 50% Tissue culture Infective Dose. HDAC1 protein expression was confirmed *via* western blotting.

### Experimental Groups and Treatments

Cell lines of H520 and H226 were carried out as independent experiments, and grouped as follows: 1) The control (control), in which cells were treated with blank solvent. 2) Tambulin treatment group (Tambulin), in which cells were treated with different doses of tambulin (diluted with medium). 3) Tambulin combined HDAC1 over-expression group (Tambulin + HDAC1), in which cells were infected with 10 MOI recombinant adenovirus containing HDAC1 gene, and then treated with tambulin after 48 h. 4) HDAC1 over-expression group (HDAC1), in which cells were infected with recombinant adenovirus containing HDAC1 gene, and then treated with blank solvent after 48 h. The MOI of adenovirus was determined during preliminary experiments.

### MTT Assay

The inhibition rates of tambulin on H226 and H520 cells were evaluated by 3-(4,5-dimethylthiazol-2-yl)-5-(3-carboxymethoxyphenyl)-2-(4-sulfophenyl)-2H-tetrazolium (MTT; Promega, WI, United States) assay. Cells were plated into 96-well plates with a density of 1 x 10^5^ cells/well. After 24 h of culture, adherent cells were treated with different doses of tambulin for 12, 24, and 48 h. Then cells were incubated with 20 μl MTT (5 mg/ml) in 100 μl cell culture medium for 4 h at 37°C. After 4 h, the absorbance of each well was measured at a wavelength of 490 nm.

### Cell Counting Assay

Sub-confluent cells were equally plated onto 6-wells-plates in complete medium for different time points as described. The media was replaced every 24 h with a fresh complete media. Cells were then trypsinized and the changes in cell counts were determined using a hemocytometer (ThermoFisher, Waltham, MA, United States).

### Apoptosis Assay

Single-cell suspensions were obtained by incubation with trypsin-EDTA for 10 min. A cell pellet was obtained by centrifugation and washed with chilled D-Hanks (pH=7.2‑7.4). The cells were then incubated in Annexin-V-APC-containing binding buffer for 15 min at room temperature. Fluorescence of Annexin-V-APC was quantified by flow cytometry (Millipore, United States) with a minimum of 10,000 cells counted for each group.

### Western Blotting

Proteins were extracted from cells by using radioimmunoprecipitation assay (RIPA) lysis buffer and size fractionated by SDS polyacrylamide gel electrophoresis. Membranes were incubated with antibodies against HDAC1 (1:1000), cleaved caspase-3 (1:1000), cleaved caspase-9 (1:1000), Bcl-2 (1:1000), Bax (1:1000), and GAPDH (1:10000) at 4°C overnight. Then membranes were incubated with the horseradish peroxidase-conjugated secondary antibodies for 2 h at room temperature after washed by tris-buffered saline and tween 20. The immune complexes were visualized by enhanced chemiluminescence after washing again and the band intensity was measured quantitatively and analyzed with the Image J v2.1.4.7 software (National Institutes of Health, United States).

### Animals

Adult male Balb/c nude mice, 4 to 6 weeks and weight 18 to 22 g, were obtained from the Nanchang University Laboratory Animal Center. The animals were allowed to access food and water ad libitum and maintained under a 12 h dark/light cycle at 22°C to 25°C. Experiments were carried out according to the Guide for the Care and Use of Laboratory Animals published by the United States. National Institutes of Health (NIH Publication No. 85 - 23, revised in 1996), and approved by the Ethics Committee of Nanchang University (No. 2019 - 0032).

### Cell Derived Xenograft (CDX)

H520 and H226 cell suspensions were prepared with a density of 1 × 10^6^ cells/ml. Applied lidocaine to the skin of the scapula of the forelegs of nude mice, then used a micro syringe to draw 100 μl of cell suspension and injected it under the skin of the scapula of the left forelimbs of nude mice. The nude mice were fed regularly and the bearing tumors were observed continuously. When the tumors grew to 100‑200 mm^3^, the mice were selected for formal experiments.

### Experimental Groups and Treatments

The mice bearing H226 and H520 CDX tumors reaching 100‑200 mm^3^ were randomly grouped with six in each group as follows: 1) The control group (Control), in which mice were oral administrated with vehicle daily for 28 days. 2) Tambulin treatment group (Tambulin), in which mice were treated orally with different doses of tambulin (20, 40, 80 mg/kg/day) for 28 days. 3) Tambulin combined HDAC1 overexpression group (Tambulin + HDAC1), in which cells were infected with recombinant adenovirus containing HDAC1 gene before being inoculated into nude mice, then mice were treated with tambulin continuously for 28 days as that in the group of Tambulin. 4) HDAC1 overexpression group (HDAC1), in which cells were infected with recombinant adenovirus containing HDAC1 gene before being inoculated into nude mice, then the mice were treated as that in the group of Control. 5) Cisplatin treatment group (CDDP), in which mice were injected intraperitoneally with 2 mg/kg cisplatin at days 1, 2, 3, and days 15, 16, 17. 6) Tambulin combined with cisplatin treatment group (Tambulin + CDDP), in which mice were treated orally with 80 mg/kg/day tambulin for 28 days and injected intraperitoneally with 2 mg/kg cisplatin at days 1, 2, 3, and days 15, 16, 17. The tumor volumes and body weights of mice in above groups were measured every 3 days. When the weight of mouse was reduced by more than 20%, or bearing tumor has ulcer or the volume was more than 2000 mm^3^, CO_2_ euthanasia would be performed. All mice were euthanized humanely on the 28^th^ day.

### Immunohistochemistry Assay

After fixing in formalin, the tumor tissue was embedded in paraffin and sectioned. Each section was immunostained using antibodies of HDAC 1 (1:100), cleaved caspase-3 (1:100), Bcl-2 (1:100), and Bax (1:10). All sections were photographed by optical microscope (Leica Microsystems, IL, United States), and analyzed with Image Pro Plus 6.0 software (Media Cybernetics, Rockville, MA, United States)

### Statistical Analysis

Data was presented as means ± standard error and analyzed by SPSS version 20.0 (IBM Corp., Armonk, NY, United States). The assumption of normality was tested with the Shapiro‑Wilk test for normality prior to additional analysis. Normally distributed data were analyzed by Student’s unpaired, two-tailed t-test (two groups), or one- or two-way ANOVA (> two groups) followed by Dunnett’s or Tukey’s post-hoc test. *P* < 0.05 was set as the level of significance.

## Results

### HDAC1 Is Highly Expressed in H226 and H520 Cells

**Figure 1:**
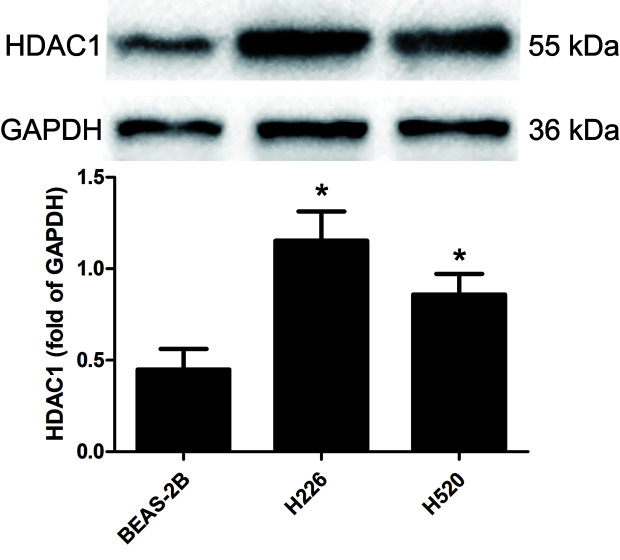
The protein expression levels of HDAC 1 in cells of BEAS-2B, H226, and H520. The values were expressed as the means ± SD (n=6 for each cell line). ^*^P < 0.05 vs. BEAS-2B.

Western blotting was used to detect the protein levels of HDAC1 in BEAS-2B, H226 and H520 cells. **Figure 1** and **Supplementary Figure 3** shows that, as compared to the non-tumorigenic BEAS-2B cell line, the protein level of HDAC1 is significantly higher in both the H226 and H520 cell lines, suggesting that HDAC1 is specifically up-regulated in LSCC.

### Effects of Tambulin on Cell Inhibition Rates of H226 and H520 Cells

**Figure 2:**
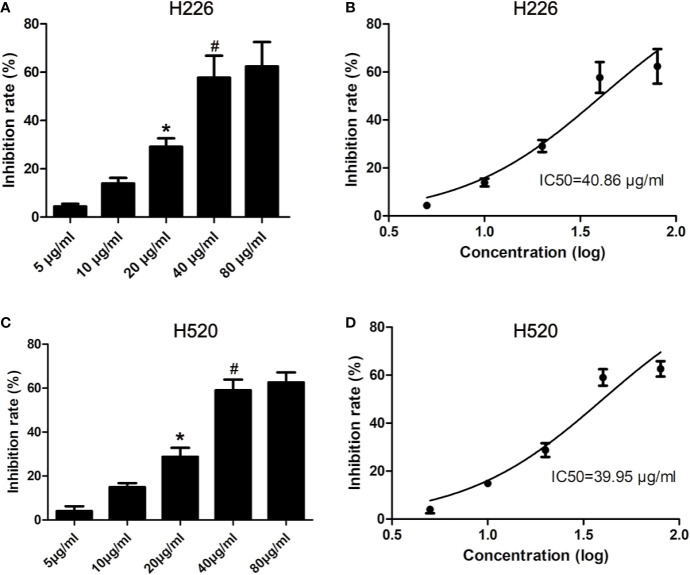
Effects of tambulin on inhibition rates of H226 and H520 cells. **(A)** Inhibition rates of different doses of tambulin on H226 cells for 24 h. **(B)** Logarithmic function dose-effect curve of tambulin on H226 cells for 24 h. **(C)** Inhibition rates of different doses of tambulin on H520 cells for 24 h. **(D)** Logarithmic function dose-effect curve of tambulin on H520 cells for 24 h. The values were expressed as the means ± SD (n=6 for each group). ^*^P <0.05 vs. 10 μg/ml; ^#^*P <* 0.05 vs. 20 μg/ml.

H226 and H520 cells were treated with different doses of tambulin and the cell inhibition rates were measured by MTT assay after 12, 24, and 48 h. As shown in **Figure 2** and **Supplementary Figure 2**, the inhibitory effects of tambulin on H226 and H520 cells showed a significant dose and time dependence. The calculated IC_50_ values of tambulin in H226 cells were 53.02, 40.86, and 36.61 μg/ml at 12, 24, and 48 h, respectively. The calculated IC_50_ values of tambulin in H520 cells were 52.71, 39.95, and 36.90 μg/ml at 12, 24, and 48 h, respectively. These results indicate that tambulin has a significant inhibitory effect in H226 and H520 cells after 24 h of treatment and the IC_50_ values of the two cell lines after a 48 h tambulin treatment is very close to that of 24 h. In all subsequent *in vitro* experiments using H226 and H520 cells a dose of 40 μg/ml was used.

### Effects of Tambulin on Cell Proliferation of H226 and H520 Cells

**Figure 3:**
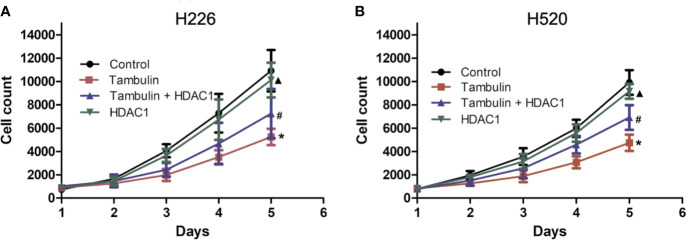
Effects of tambulin on cell proliferation of H226 and H520 cells. The doses of tambulin here were always 40 μg/ml in H226 and H520 cells. **(A)** Cell proliferation curve of H226. **(B)** Cell proliferation curve of H520. The values were expressed as the means ± SD (n=6 for each group). ^*^P < 0.05 vs. Control; ^#^*P <* 0.05 vs. Tambulin; ^▲^*P <* 0.05 vs. Tambulin + HDAC1.

Cell proliferation was measured by cell counts. As shown in **Figure 3**, tambulin significantly reduced the proliferation of H226 or H520 cells as compared to the control group (*P* < 0.05). However, in the experimental group of Tambulin + HDAC1, the proliferation of H226 and H520 cells increased significantly as compared to tambulin treatment only (*P* < 0.05). Furthermore, proliferation of H226 and H520 cells was significantly increased in the HDAC1 overexpression group as compared to the Tambulin + HDAC1 group (*P* < 0.05), suggesting that HDAC1 overexpression is able to rescue the anti-proliferative effects of tambulin treatment.

### Effects of Tambulin on the Apoptosis of H226 and H520 Cells

**Figure 4:**
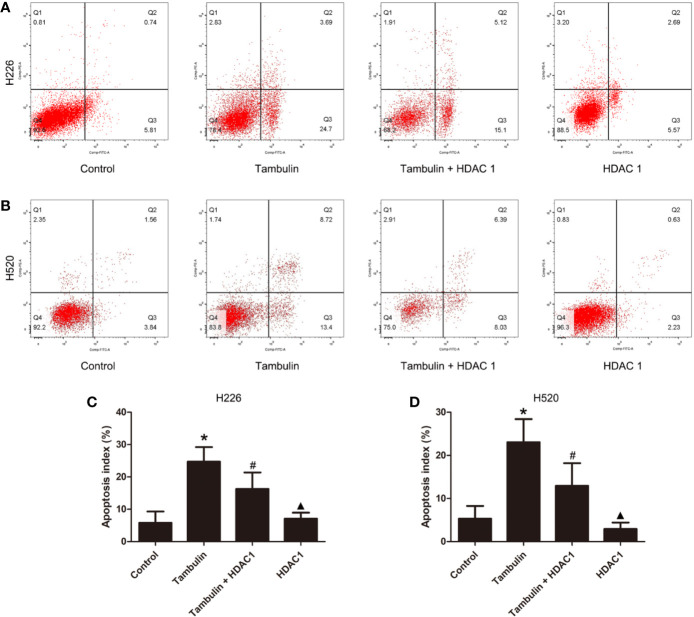
Effects of tambulin on apoptosis of H226 and H520 cells. The doses of tambulin here were always 40 μg/ml in H226 and H520 cells. **(A)** Representative images of flow cytometry in each experimental group of H226 cell line. **(B)** Representative images of flow cytometry in each experimental group of H520 cell line. **(C)** Apoptosis rate in each experimental group of H226 cell line. **(D)** Apoptosis rate in each experimental group of H520 cell line. The values were expressed as the means ± SD (n = 6 for each group). ^*^P < 0.05 vs. Control; ^#^*P <* 0.05 vs. Tambulin; ^▲^*P <* 0.05 vs. Tambulin + HDAC1.

The apoptotic rate of each experimental group was detected by flow cytometry. As shown in **Figure 4**, the number of H226 and H520 cells undergoing apoptosis was significantly increased in the tambulin treatment group as compared to the control group (*P* < 0.05), while overexpression of HDAC1 significantly inhibited the apoptotic rate of H226 and H520 as compared to the tambulin only group (*P* < 0.05). The apoptotic rate of H226 and H520 cells in the HDAC1 overexpression group was significantly decreased as compared to the Tambulin + HDAC1 group (*P* < 0.05).

### Effects of Tambulin on the Expression Level of HDAC1 in H226 and H520 Cells

**Figure 5:**
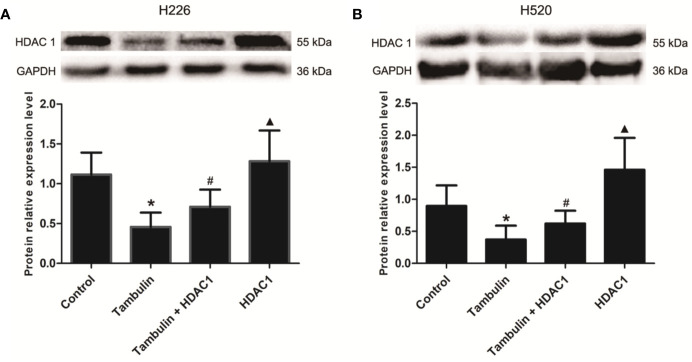
Effects of tambulin on the expression levels of HDAC 1 in H226 and H520 cells. The doses of tambulin here were always 40 μg/ml in H226 and H520 cells. **(A)** The expression level of HDAC1 in H226 cells. **(B)** The expression level of HDAC1 in H520 cells. The values were expressed as the means ± SD (n=6 for each group). ^*^P < 0.05 vs. Control; ^#^*P <* 0.05 vs. Tambulin; ^▲^*P <* 0.05 vs. Tambulin + HDAC1.

Western blotting was used to detect the protein levels of HDAC1 in each experimental group. As shown in **Figure 5** and **Supplementary Figure 4**, the protein levels of HDAC1 were significantly down-regulated after tambulin treatment in H226 and H520 cells as compared to the control group (*P* < 0.05), while transduction of H226 and H520 cells with recombinant adenovirus successfully up-regulated HDAC1 (*P* < 0.05, Tambulin group vs. Tambulin + HDAC1 group). In addition, overexpressing HDAC1 without tambulin intervention significantly up-regulated the protein expression of HDAC1 in H226 and H520 cells as compared to the Tambulin + HDAC1 group (*P* < 0.05).

### Effects of Tambulin on the Expression Levels of Apoptosis Related Proteins in H226 and H520 Cells

**Figure 6:**
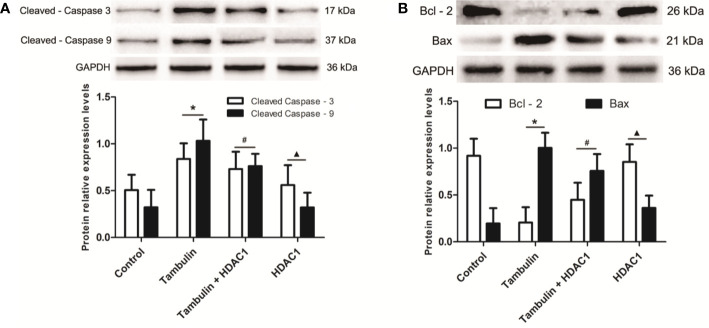
Effects of tambulin on the expression levels of cleaved caspase-3, cleaved caspase-9, Bcl-2, and Bax in H226 cells. The dose of tambulin here was 40 μg/ml in H226 cells. **(A)** The expression levels of cleaved caspase-3 and cleaved caspase-9 in H226 cells. **(B)** The expression levels of Bcl-2 and Bax in H226 cells. The values were expressed as the means ± SD (n = 6 for each group). ^*^P < 0.05 vs. Control; ^#^*P <* 0.05 vs. Tambulin; ^▲^*P <* 0.05 vs. Tambulin + HDAC1.

**Figure 7:**
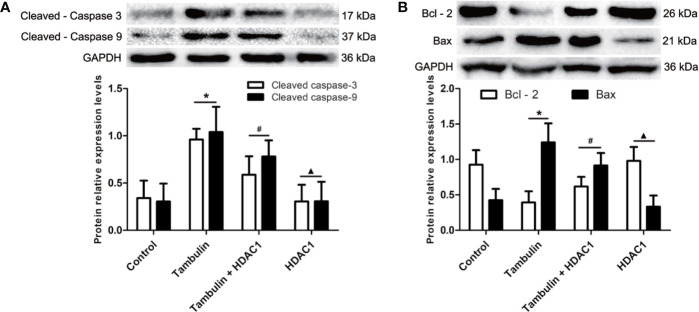
Effects of tambulin on the expression levels of cleaved caspase-3, cleaved caspase-9, Bcl-2 and Bax in H520 cells. The dose of tambulin here was 40 μg/ml in H520 cells. **(A)** The expression levels of cleaved caspase-3 and cleaved caspase-9 in H520 cells. **(B)** The expression levels of Bcl-2 and Bax in H520 cells. The values were expressed as the means ± SD (n=6 for each group). ^*^P < 0.05 vs. Control; ^#^*P <* 0.05 vs. Tambulin; ^▲^*P <* 0.05 vs. Tambulin + HDAC1.

Western blotting was used to detect the expression levels of cleaved caspase-3, cleaved caspase-9, Bcl-2, and Bax in H226 or H520 cells. As shown in **Figures 6** and **7** (**Supplementary Figures 5** and **6**), the protein expressions of cleaved caspase-3, cleaved caspase-9, and Bax were up-regulated, and the Bcl-2 expression was down-regulated significantly in H226 or H520 cells after the treatment of tambulin comparing with the control group (P < 0.05). Overexpression HDAC1 could reverse the effects of tambulin on the expression levels of cleaved caspase-3, cleaved caspase-9, Bcl-2, or Bax obviously in H226 or H520 cells (P < 0.05 vs. the Tambulin groups). When comparing with the groups of Tambulin + HDAC1, the protein expressions of cleaved caspase-3, cleaved caspase-9, and Bax were down-regulated, and the Bcl-2 was up-regulated remarkably in the groups of HDAC 1 (P < 0.05).

### Effects of Tambulin on CDX Tumor Volume

**Figure 8:**
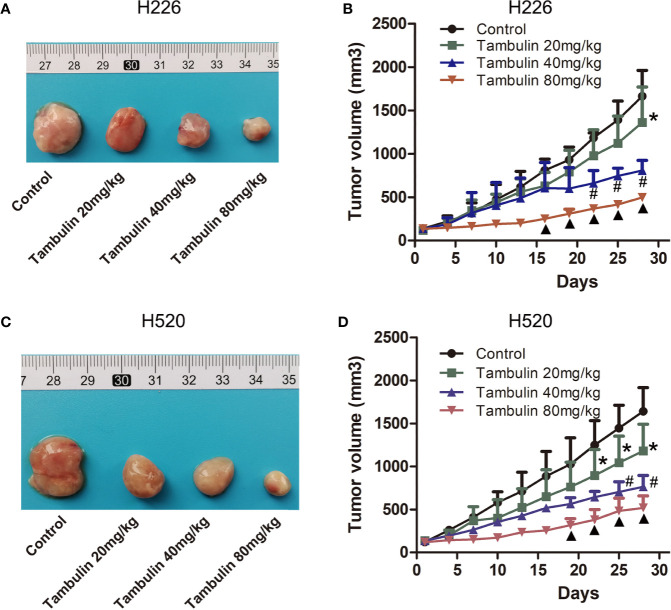
Effects of different doses of tambulin on tumor volume of CDX. **(A)** Representative image of bearing tumors of H226 in each experimental group. **(B)** Growth curves of bearing tumors of H226 in each experimental group. **(C)** Representative image of bearing tumors of H520 in each experimental group. **(D)** Growth curves of bearing tumors of H520 in each experimental group. The values were expressed as the means ± SD (n=6 for each group). ^*^P < 0.05 vs. Control; ^#^*P <* 0.05 vs. Tambulin 20 mg/kg; ^▲^*P <* 0.05 vs. Tambulin 40 mg/kg.

The effects of different doses of tambulin on mice bearing H226 and H520 CDX tumors are shown in **Figure 8**. Different doses of tambulin treatment showed definite inhibitory effects on H226 and H520 CDX tumors. The reduction in tumor growth was dose dependent. A tambulin dose of 80 mg/kg was most effective at inhibiting tumor growth in both CDX models.

**Figure 9:**
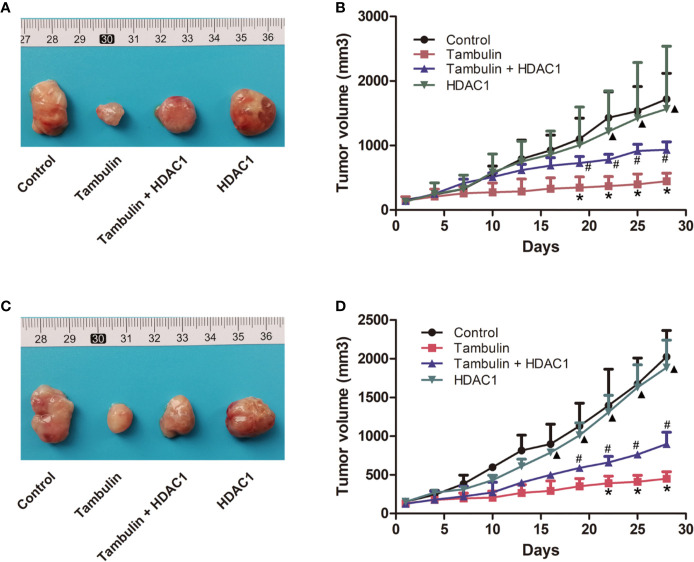
Effects of HDAC1 overexpression on tumor volume of CDX. The dose of tambulin here was 80 mg/kg. **(A)** Representative image of bearing tumors of H226 in each experimental group. **(B)** Growth curves of bearing tumors of H226 in each experimental group. **(C)** Representative image of bearing tumors of H520 in each experimental group. **(D)** Growth curves of bearing tumors of H520 in each experimental group. The values were expressed as the means ± SD (n=6 for each group). ^*^P < 0.05 vs. Control; ^#^*P <* 0.05 vs. Tambulin; ^▲^*P <* 0.05 vs. Tambulin + HDAC1.

Interestingly, as shown in **Figure 9**, overexpression of HDAC1 significantly reversed the effects of tambulin on inhibiting the growth of H226 and H520 CDX tumors (*P* < 0.05 vs. Tambulin group). In addition, the growth of H226 or H520 CDX tumors in HDAC1 overexpression group showed no significantly difference with that in control group (*P* > 0.05).

In addition, by monitoring the weight changes of nude mice, we found that tambulin intervention and/or HDAC1 overexpression had no significant effect on the weights of nude mice compared with that in the control group (*P* < 0.05) (**Tables 1** and **2**).

**Table 1:** Effects of different doses of tambulin treatment on body weights of nude mice (Mean ± S.E.M).

Cell line	Groups	n	Body weight(g)									
			1 day	4 days	7 days	10 days	13 days	16 days	19 days	22 days	25 days	28 days
	Control	6	21.31 ± 0.54	21.29 ± 0.47	21.89 ± 0.62	22.82 ± 0.65	22.75 ± 0.63	23.27 ± 0.59	23.36 ± 0.55	23.64 ± 0.51	23.39 ± 0.46	23.75 ± 0.53
H226	Tambulin 20 mg/kg	6	20.67 ± 0.32	20.45 ± 0.57	21.33 ± 0.41	21.52 ± 0.54	21.61 ± 0.47	22.32 ± 0.48	22.78 ± 0.43	22.89 ± 0.63	22.81 ± 0.63	23.13 ± 0.49
	Tambulin 40 mg/kg	6	20.47 ± 0.64	20.32 ± 0.50	21.25 ± 0.80	21.57 ± 0.64	21.13 ± 0.73	21.88 ± 0.88	21.92 ± 0.65	22.04 ± 0.55	22.71 ± 0.66	22.78 ± 0.73
	Tambulin 80 mg/kg	6	21.17 ± 0.29	21.37 ± 0.59	20.89 ± 0.79	20.47 ± 0.43	20.72 ± 0.58	21.34 ± 0.59	21.46 ± 0.58	21.75 ± 0.64	21.55 ± 0.83	21.72 ± 1.01
	Control	6	19.81 ± 0.82	20.61 ± 0.47	21.14 ± 0.79	21.45 ± 0.68	22.05 ± 0.88	22.34 ± 0.71	22.45 ± 0.74	23.11 ± 0.82	23.56 ± 0.69	23.47 ± 0.77
H520	Tambulin 20 mg/kg	6	20.12 ± 0.60	20.35 ± 0.73	20.98 ± 0.54	21.08 ± 0.76	21.60 ± 0.93	21.87 ± 0.83	22.45 ± 0.59	22.76 ± 0.62	22.72 ± .067	23.02 ± 0.64
	Tambulin 40 mg/kg	6	19.73 ± 0.68	19.90 ± 0.84	20.52 ± 0.87	20.36 ± 0.83	21.10 ± 0.77	21.44 ± 0.58	21.76 ± 0.79	22.05 ± 0.74	22.25 ± 0.80	22.57 ± 0.69
	Tambulin 80 mg/kg	6	19.60 ± 1.01	19.50 ± 0.88	19.88 ± 0.91	20.86 ± 0.93	20.47 ± 0.94	20.84 ± 0.83	21.51 ± 0.79	21.04 ± 0.76	21.80 ± 0.50	21.62 ± 0.64

**Table 2:** Effects of HDAC1 overexpression on body weights of nude mice (Mean ± S.E.M).

Cell line	Groups	n	Body weight(g)									
			1 day	4 days	7 days	10 days	13 days	16 days	19 days	22 days	25 days	28 days
	Control	6	20.48 ± 0.73	21.34 ± 0.65	21.28 ± 0.83	21.13 ± 0.37	22.42 ± 0.76	22.26 ± 0.82	23.11 ± 0.71	23.23 ± 0.47	23.26 ± 0.52	23.56 ± 0.58
H226	Tambulin	6	21.42 ± 0.57	21.47 ± 0.61	20.87 ± 0.54	20.75 ± 0.77	21.31 ± 0.63	21.15 ± 0.72	21.92 ± 0.31	22.44 ± 0.76	22.43 ± 0.54	22.81 ± 0.66
	Tambulin + HDAC1	6	20.41 ± 0.58	20.24 ± 0.36	20.62 ± 0.65	21.52 ± 0.61	21.84 ± 0.74	21.82 ± 0.55	22.16 ± 0.44	22.42 ± 0.58	22.47 ± 0.39	22.65 ± 0.41
	HDAC1	6	20.63 ± 0.78	20.56 ± 1.01	21.12 ± 0.74	21.54 ± 0.66	21.13 ± 0.77	21.83 ± 0.42	22.42 ± 0.48	22.72 ± 0.26	23.08 ± 0.48	23.16 ± 0.20
	Control	6	20.15 ± 0.46	20.95 ± 0.88	20.45 ± 0.62	20.94 ± 0.58	21.34 ± 0.46	21.21 ± 0.43	21.78 ± 0.40	22.33 ± 0.49	22.67 ± 0.62	23.16 ± 0.58
H520	Tambulin	6	19.87 ± 0.32	19.62 ± 0.47	19.90 ± 0.55	20.63 ± 0.45	20.10 ± 0.61	20.72 ± 0.66	20.88 ± 0.60	21.51 ± 0.72	21.63 ± 0.77	21.73 ± 0.78
	Tambulin + HDAC1	6	20.40 ± 0.63	20.05 ± 0.71	20.43 ± 0.58	20.85 ± 0.66	21.13 ± 0.67	21.40 ± 0.74	21.35 ± 0.53	21.83 ± 0.31	21.67 ± 0.69	22.04 ± 0.55
	HDAC1	6	20.56 ± 0.54	20.46 ± 0.57	20.84 ± 0.51	21.06 ± 0.71	21.39 ± 0.81	21.39 ± 0.68	21.92 ± 0.42	22.35 ± 0.59	22.62 ± 0.59	22.88 ± 0.47

### Effects of Tambulin on the *In Vivo* Expression Levels of HDAC1 and Apoptosis Related Proteins

**Figure 10:**
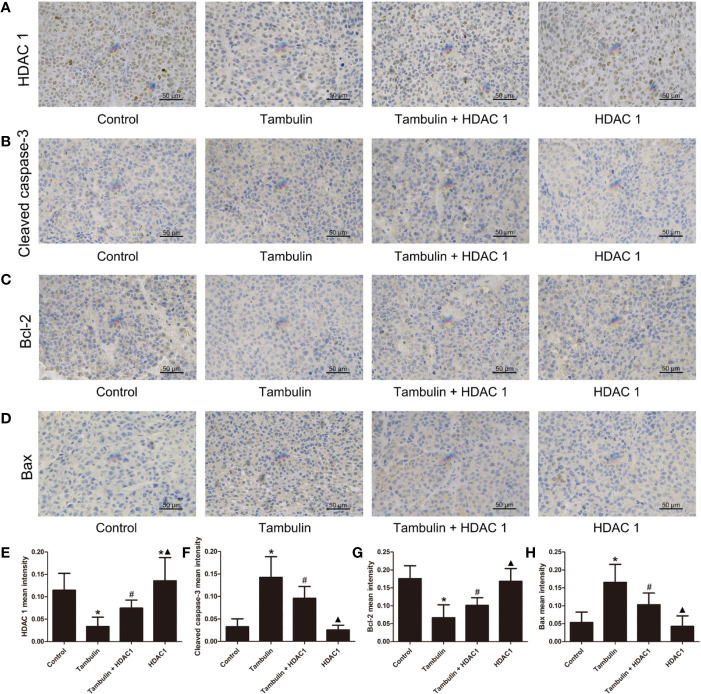
Effects of tambulin on the protein expression levels of HDAC 1, cleaved caspase-3, Bcl-2 and Bax in bearing tumors of H226. The dose of tambulin here was 80 mg/kg. **(A)** Representative immunohistochemical images of HDAC1 in each experimental group. **(B)** Representative immunohistochemical images of cleaved caspase-3 in each experimental group. **(C)** Representative immunohistochemical images of Bcl-2 in each experimental group. **(D)** Representative immunohistochemical images of Bax in each experimental group. **(E)** HDAC 1 mean intensity of each experimental group. **(F)** Cleaved caspase-3 mean intensity of each experimental group. **(G)** Bcl-2 mean intensity of each experimental group. **(H)** Bax mean intensity of each experimental group. The values were expressed as the means ± SD (n=6 for each group). ^*^P < 0.05 vs. Control; ^#^*P <* 0.05 vs. Tambulin; ^▲^*P <* 0.05 vs. Tambulin + HDAC1.

**Figure 11:**
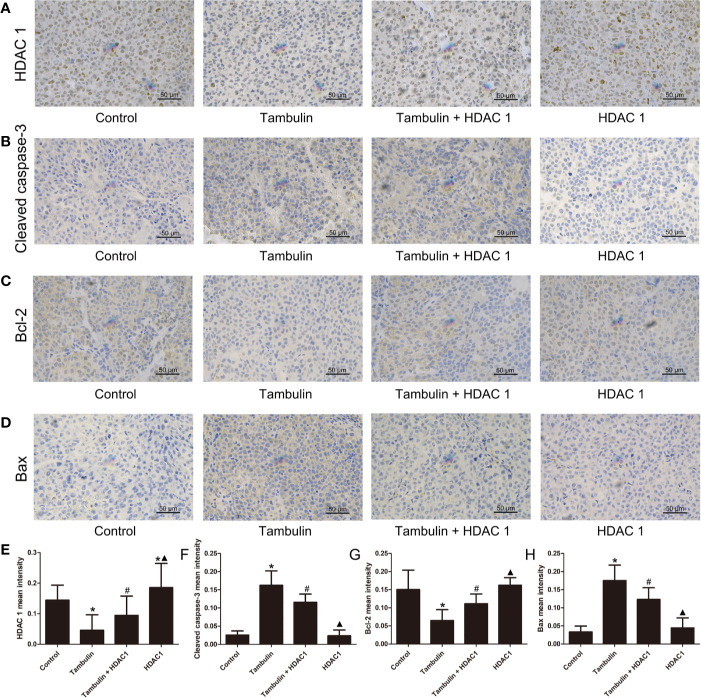
Effects of tambulin on the protein expression levels of HDAC1, cleaved caspase-3, Bcl-2 and Bax in bearing tumors of H520. The dose of tambulin here was 80 mg/kg. **(A)** Representative immunohistochemical images of HDAC1 in each experimental group. **(B)** Representative immunohistochemical images of cleaved caspase-3 in each experimental group. **(C)** Representative immunohistochemical images of Bcl-2 in each experimental group. **(D)** Representative immunohistochemical images of Bax in each experimental group. **(E)** HDAC1 mean intensity of each experimental group. **(F)** Cleaved caspase-3 mean intensity of each experimental group. **(G)** Bcl-2 mean intensity of each experimental group. **(H)** Bax mean intensity of each experimental group. The values were expressed as the means ± SD (n=6 for each group). ^*^P < 0.05 vs. Control; ^#^*P <* 0.05 vs. Tambulin; ^▲^*P <* 0.05 vs. Tambulin + HDAC1.

Immunohistochemistry was used to detect the expression levels of HDAC1, cleaved caspase-3, Bcl-2 and Bax in H226 and H520 CDXs tumor cells. As shown in **Figures 10** and **11**, continuous tambulin intervention significantly down-regulated the expression of HDAC1 and Bcl-2, and simultaneously up-regulated the expression of cleaved caspase-3 and Bax in H226 and H520 CDX tumors (*P* < 0.05 vs. the control group). However interestingly, the effects of tambulin on the expression levels of HDAC1, cleaved caspase-3, cleaved caspase-9, Bcl-2, and Bax were all reversed when HDAC1 was overexpressed (*P* < 0.05 vs. Tambulin group). Additionally, in the HDAC1 overexpression group, the expression of HDAC1 and Bcl-2 were up-regulated, and the expression of cleaved caspase-3 and Bax were down-regulated in H226 and H520 CDX tumors as compared to the Tambulin + HDAC1 group (*P* < 0.05).

### Effects of Tambulin and Cisplatin on CDX Tumor Volume

**Figure 12:**
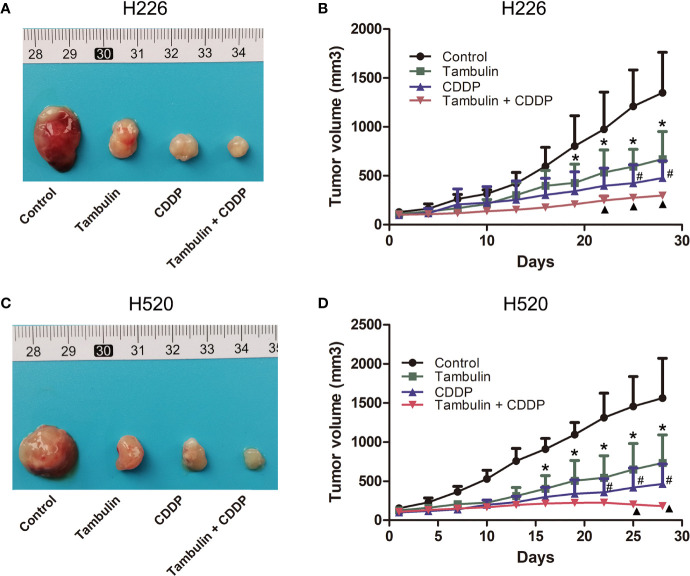
Effects of tambulin and cisplatin on tumors volume of CDX. The dose of tambulin here was 80 mg/kg, and cisplatin was 2 mg/kg. **(A)** Representative image of bearing tumors of H226 in each experimental group. **(B)** Growth curves of bearing tumors of H226 in each experimental group. **(C)** Representative image of bearing tumors of H520 in each experimental group. **(D)** Growth curves of bearing tumors of H520 in each experimental group. The values were expressed as the means ± SD (n=6 for each group). ^*^P < 0.05 vs. Control; ^#^*P <* 0.05 vs. Tambulin; ^▲^*P <* 0.05 vs. CDDP.

As shown in **Figure 12**, both tambulin and cisplatin significantly inhibited the growth of H226 and H520 CDX tumors as compared to the control group (*P* < 0.05). As expected, cisplatin alone was more effective than tambulin alone (*P* < 0.05). Interestingly, the combination of tambulin and cisplatin was more effective than tambulin or cisplatin alone in H226 and H520 CDX (*P* < 0.05) suggesting that tambulin could enhance the anti-tumor effect of cisplatin. What’s more, as shown in **Table 3**, tambulin and/or cisplatin intervention had no significant effect on the weights of nude mice compared with that in the control group (*P* < 0.05).

**Table 3:** Effects of tambulin and cisplatin on body weights of nude mice (Mean ± S.E.M).

Cell line	Groups	n	Body weight(g)									
			1 day	4 days	7 days	10 days	13 days	16 days	19 days	22 days	25 days	28 days
	Control	6	21.63 ± 0.33	21.07 ± 0.55	22.19 ± 0.56	22.16 ± 0.47	22.79 ± 0.99	22.93 ± 0.87	23.42 ± 0.63	23.86 ± 0.67	23.83 ± 0.40	23.78 ± 0.51
H226	Tambulin	6	21.12 ± 0.57	21.66 ± 0.45	22.28 ± 0.54	22.39 ± 0.49	22.65 ± 0.47	22.72 ± 0.58	22.87 ± 0.51	23.25 ± 0.65	23.20 ± 0.82	23.25 ± 0.62
	CDDP	6	20.65 ± 0.27	20.92 ± 0.51	21.26 ± 0.64	21.86 ± 0.59	22.47 ± 0.73	22.17 ± 0.48	21.35 ± 0.63	21.68 ± 0.74	22.22 ± 0.77	22.38 ± 0.81
	Tambulin + CDDP	6	21.86 ± 0.66	20.67 ± 0.69	20.63 ± 0.90	20.29 ± 1.07	21.65 ± 1.15	21.60 ± 1.07	21.53 ± 1.31	21.67 ± 1.26	21.33 ± 1.24	21.13 ± 1.24
	Control	6	19.84 ± 0.42	20.18 ± 0.38	20.30 ± 0.68	20.72 ± 0.47	21.17 ± 0.55	21.49 ± 0.60	21.99 ± 0.62	22.27 ± 00.68	22.50 ± 0.37	22.78 ± 0.72
H520	Tambulin	6	20.19 ± 0.33	20.58 ± 0.21	20.68 ± 0.34	20.52 ± 0.71	20.86 ± 0.92	21.15 ± 0.42	21.26 ± 0.69	21.58 ± 0.73	21.88 ± 0.64	21.86 ± 0.55
	CDDP	6	19.52 ± 0.29	19.47 ± 0.24	19.82 ± 0.39	20.47 ± 0.54	20.52 ± 0.41	20.78 ± 0.53	21.22 ± 0.61	21.73 ± 0.73	21.67 ± 0.43	22.01 ± 0.69
0.69	Tambulin + CDDP	6	20.21 ± 0.37	20.33 ± 0.44	20.19 ± 0.65	20.58 ± 0.57	20.74 ± 0.52	21.29 ± 0.74	21.08 ± 0.66	21.22 ± 0.57	21.34 ± 0.49	21.42 ± 0.53

## Discussion

Tambulin is extracted from *Zanthoxylum armature*. Nooreen *et al*. analyzed the biological activity of various extracts of *Zanthoxylum bungeanum* and found that, when compared to other extracts, tambulin had a more anti-proliferative effect on A549, WRL-68, HaCaT, MCF-7, MDA-MB231, and color-205 tumor cell lines and thus classified it as a potential anti-cancer drug (Nooreen et al., 2017). In present study, for the first time, we showed that tambulin could significantly inhibit the proliferation of H520 and H226 cells, two LSCC cell lines. Tambulin intervention can also significantly inhibit tumor growth of H226 and H520 CDXs. The above results show that tambulin has a marked therapeutic effect on LSCC.

Apoptosis is an important process by which the body maintains homeostasis. Caspases belong to the cysteine aspartate-specific protease family and are the main executors of apoptosis. The abnormal regulation of caspases is closely related to the occurrence and development of cancer (Looi et al., 2013). The caspase family is divided into initiating caspases (such as caspase-8, -9, and 10) and effector caspases (such as caspases-3, -6, and -7). Activation of caspase-3 and -7 is essential to induce downstream DNA cleavage molecules (Wu et al., 2014). Activating caspases is an effective strategy to induce cancer cell apoptosis. The Bcl-2/Bax pathway plays a key role in endogenous apoptosis. As an inhibitor of apoptosis, Bcl-2 exerts anti-apoptotic effects by counteracting pro-apoptotic proteins produced by Bax which in turn permeabilize the outer membrane of mitochondria, inhibiting the release of cytochrome *c* to the cytoplasm and suppressing the cytochrome *c* mediated caspase cascade (Kontos et al., 2014). In this study, we found that tambulin significantly promotes the apoptosis of H266 and H520 cells *in vivo*. It also up-regulates the expression of pro-apoptotic factors including cleaved caspase-3, cleaved caspase-9 and Bax, and down-regulates the expression of Bcl-2, an inhibitor of apoptosis. These results indicate that the anti-LSCC mechanism of tambulin is mediated by regulating the Bcl-2/caspase pathway to induce apoptosis of cancer cells.

Histone acetylation and deacetylation are important parts of gene expression regulation. Histone deacetylation is regulated by histone deacetylases (HDACs), which can inhibit DNA methyltransferase through DNA methylation (Roche and Bertrand, 2016). Based on sequence homology, the HDAC family is divided into four categories, namely I, IIa/IIb, III, and IV. Among them, HDAC1, a class I HDAC, plays an important role in regulating DNA damage signals to maintain genomic stability and enhance tumor malignancy *in vivo* (Thurn et al., 2013; Stojanovic et al., 2016; Huang et al., 2018). HDAC1 is highly expressed in various tumors including lung carcinoma. Knock-out of the *HDAC1* gene causes cell cycle arrest, a decrease in cell survival rate and an increase in apoptosis (Selokar et al., 2013). High expression of HDAC1 increases invasion and proliferation of glioma cells which promotes the progression and recurrence of glioma tumors (Wang et al., 2017). A meta-analysis showed that the expression level of HDAC1 was closely correlated to the progression and prognosis of lung carcinoma and could be used as a diagnostic and prognostic indicator of lung carcinoma (Cao et al., 2017). Recent studies have shown that HDAC1 expression is increased in NSCLC. Knock-down of HDAC1 decreases the viability, migration, invasion, and angiogenesis, while increasing apoptosis in NSCLC cells (Zhang et al., 2018). Therefore, it is believed that HDAC1 is a promoter of NSCLC (Zhang et al., 2018). In this study, we show that HDAC1 protein levels are significantly higher in H226 and H520 cells as compared to the normal lung cell line, BEAS-2B. Tambulin treatment significantly decreases the protein expression of HDAC1 in H5226 and H520 cells. Tambulin treatment also resulted in reduced proliferation and increased apoptosis of H226 and H520 cells. Interestingly however, the effects of tambulin on cell proliferation and apoptosis were attenuated after HDAC1 over-expression. Additionally, the effects of tambulin on the protein expression of cleaved caspase-3, cleaved caspase-9, Bcl-2, and Bax were also significantly reversed. The above results show that tambulin down-regulates HDAC1 expression. In turn, this deregulates the Bcl-2/caspase pathway and promotes apoptosis of LSCC cells.

In recent decades, many HDAC inhibitors have been developed for anti-cancer purposes. HDAC inhibitors can target a variety of subtypes of HDAC enzymes in a broad or selective manner, affect multiple aspects of tumor development and thus exert anti-tumor effects (Carew et al., 2008). However, the efficacy of HDAC inhibitors alone in solid tumors has so far been disappointing (Sun et al., 2018). Encouraging results have been achieved through the combination of HDAC inhibitors and other drugs. The study of Ramalingam *et al*. showed that, although there was no significant change in improving the progression-free survival or overall survival, paclitaxel and carboplatin combined with the HDAC inhibitor vorinostat could significantly improve the clinical response of patients with NSCLC (Ramalingam et al., 2010). HDAC inhibitors can re-sensitize metastatic lung carcinoma to EGFR inhibitors (Witta et al., 2006). Kong *et al*. showed that the HDAC inhibitor belinostat can enhance the sensitivity of cisplatin in LSCC (Kong et al., 2017). In this study, we showed that a combination of tambulin and cisplatin inhibited the growth of LSCC CDXs more significantly than tambulin or cisplatin alone. Therefore, we believe that tambulin combined with cisplatin will obtain a better anti-LSCC effect and tambulin may sensitize LSCC to cisplatin.

In conclusion, using *in vivo* and *in vitro* studies, we observed that tambulin exhibits a definite anti-cancer effect on LSCC. Mechanistically, tambulin targets HDAC1, thereby regulating the Bcl-2/caspase signaling pathway which ultimately induces apoptosis of LSCC cells. Interestingly, tambulin combined with cisplatin showed a more efficacious anti-LSCC effect, suggesting that tambulin sensitizes LSCC to cisplatin. The results presented here indicate that tambulin is a potential anti-LSCC drug and has a definite developmental value, however, further research is required to fully demonstrate the scope of its anti-cancer potential.

## Data Availability Statement

The raw data supporting the conclusions of this article will be made available by the authors, without undue reservation.

## Ethics Statement

The animal study was reviewed and approved by Ethics Committee of Nanchang University.

## Author Contributions

WW and YL contributed equally to this work. All authors contributed to the article and approved the submitted version.

## Funding

This study was supported by the Science and Technology Plan of Jiangxi Provincial Chest Hospital (grant no. 202042).

## Conflict of Interest

The authors declare that the research was conducted in the absence of any commercial or financial relationships that could be construed as a potential conflict of interest.

## Supplementary Material

Supplementary Figure 1 Chemical structure of tambulin (PubChem CID: 5281700).

Supplementary Figure 2 Effects of tambulin on inhibition rates of H226 and H520 cells. **(A)** Inhibition rates of different doses of tambulin on H226 cells for 12 h. **(B)** Logarithmic function dose-effect curve of tambulin on H226 cells for 12 h. **(C)** Inhibition rates of different doses of tambulin on H226 cells for 48 h. **(D)** Logarithmic function dose-effect curve of tambulin on H226 cells for 48 h. **(E)** Inhibition rates of different doses of tambulin on H520 cells for 12 h. **(F)** Logarithmic function dose-effect curve of tambulin on H520 cells for 12 h. **(G)** Inhibition rates of different doses of tambulin on H520 cells for 48 h. **(H)** Logarithmic function dose-effect curve of tambulin on H520 cells for 48 h. The values were expressed as the means ± SD (n=6 for each group). ^*^*P <*0.05 vs. 10 μg/ml; ^#^*P <*0.05 vs. 20 μg/ml.

Supplementary Figure 3 The uncropped western blots of **Figure 1**. **(A)** The uncropped western blots of HDAC1. **(B)** The uncropped western blots of GAPDH.

Supplementary Figure 4 The uncropped western blots of **Figure 5**. **(A)** The uncropped western blots of HDAC1 in H226. **(B)** The uncropped western blots of GAPDH in H226. **(C)** The uncropped western blots of HDAC1 in H520. **(D)** The uncropped western blots of GAPDH in H520.

Supplementary Figure 5 The uncropped western blots of **Figure 6**. **(A)** The uncropped western blots of cleaved-caspase 3 in H226 cells. **(B)** The uncropped western blots of cleaved-caspase 9 in H226 cells. **(C)** The uncropped western blots of GAPDH in H226 cells. **(D)** The uncropped western blots of Bcl-2 in H226 cells. **(E)** The uncropped western blots of Bax in H226 cells. **(F)** The uncropped western blots of GAPDH in H226 cells.

Supplementary Figure 6 The uncropped western blots of **Figure 7**. **(A)** The uncropped western blots of cleaved-caspase 3 in H520 cells. **(B)** The uncropped western blots of cleaved-caspase 9 in H520 cells. **(C)** The uncropped western blots of GAPDH in H520 cells. **(D)** The uncropped western blots of Bcl-2 in H520 cells. **(E)** The uncropped western blots of Bax in H520 cells. **(F)** The uncropped western blots of GAPDH in H520 cells.
