# Supplementary figures and images for: Tambulin Targets Histone Deacetylase 1 Inhibiting Cell Growth and Inducing Apoptosis in Human Lung Squamous Cell Carcinoma
Source: Front Pharmacol. 2020 Aug 12;11:1188. doi: 10.3389/fphar.2020.01188 (PMC7434869; doi:10.3389/fphar.2020.01188)

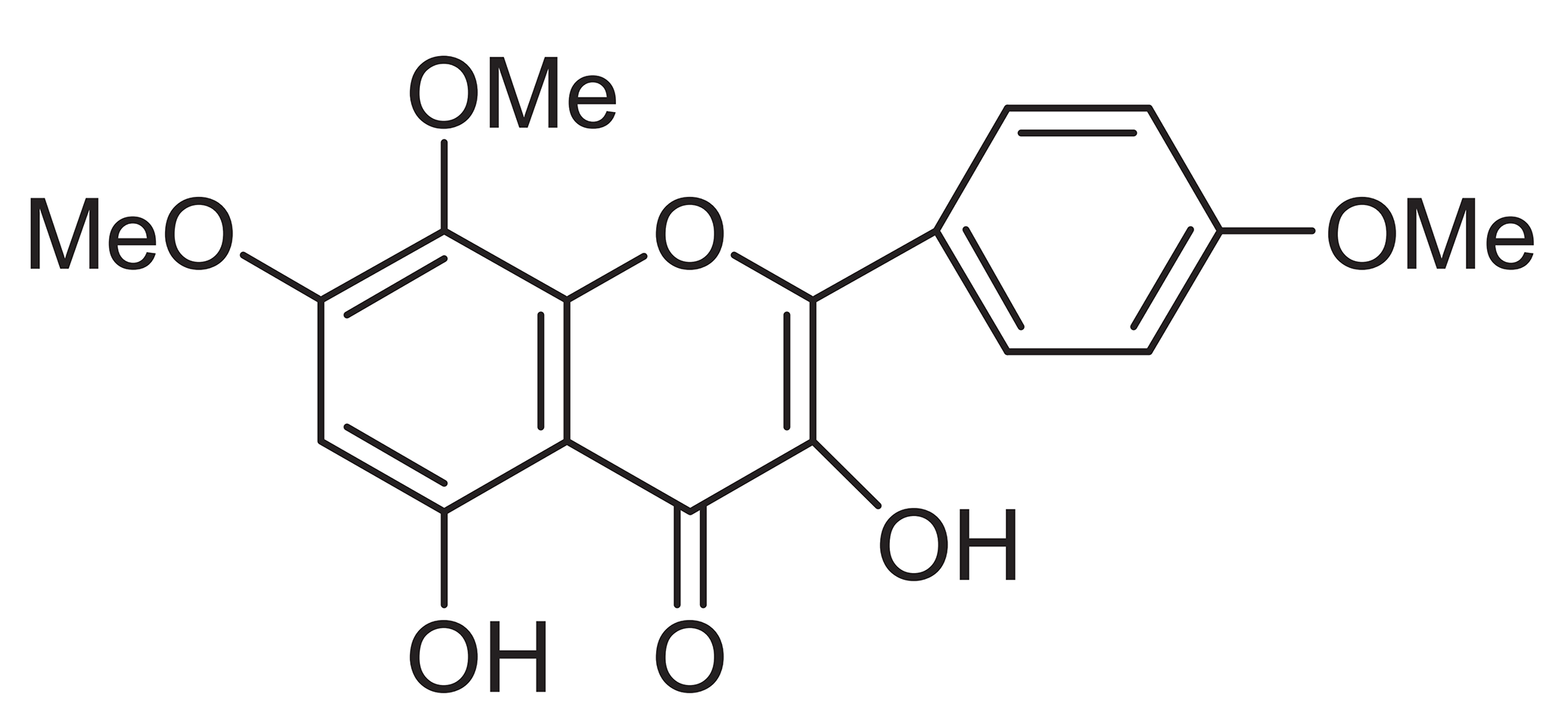

Supplement: Supplementary Figure 1 — Chemical structure of tambulin (PubChem CID: 5281700). [file Image_1.tif]

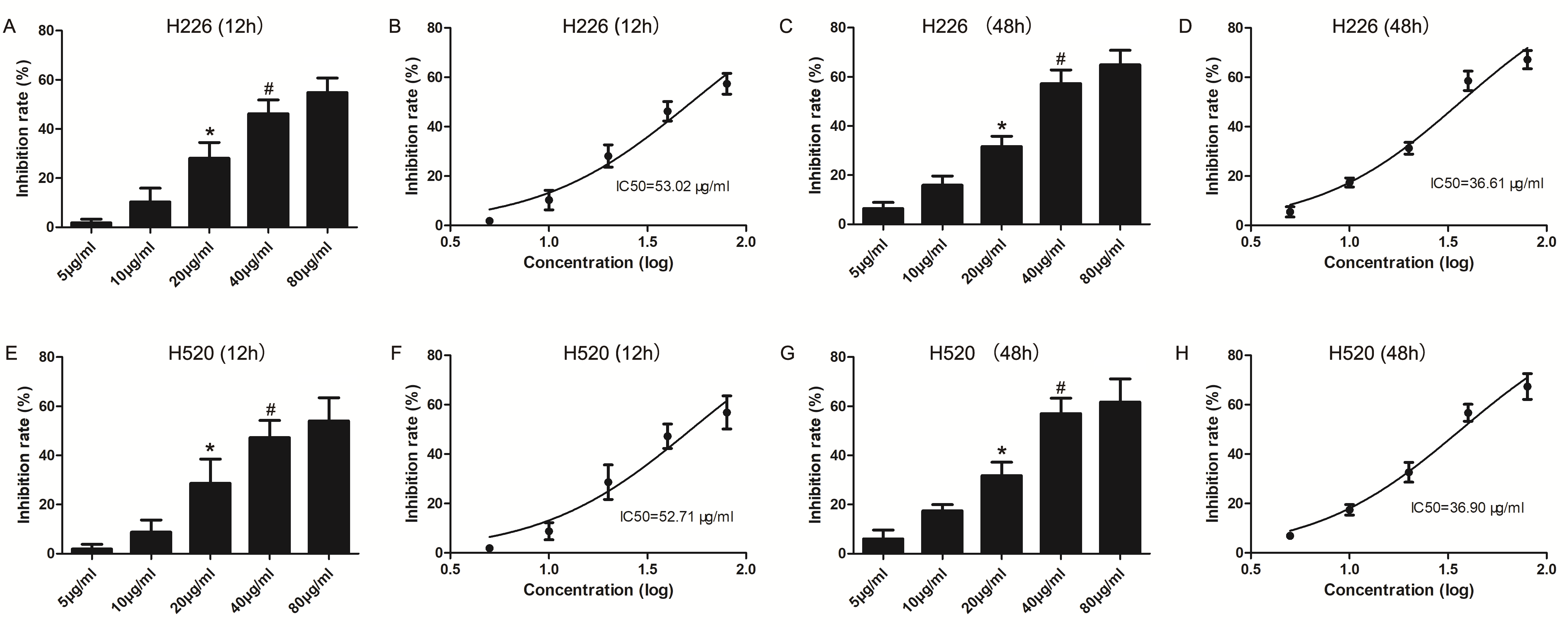

Supplement: Supplementary Figure 2 — Effects of tambulin on inhibition rates of H226 and H520 cells. (A) Inhibition rates of different doses of tambulin on H226 cells for 12 h. (B) Logarithmic function dose-effect curve of tambulin on H226 cells for 12 h. (C) Inhibition rates of different doses of tambulin on H226 cells for 48 h. (D) Logarithmic function dose-effect curve of tambulin on H226 cells for 48 h. (E) Inhibition rates of different doses of tambulin on H520 cells for 12 h. (F) Logarithmic function dose-effect curve of tambulin on H520 cells for 12 h. (G) Inhibition rates of different doses of tambulin on H520 cells for 48 h. (H) Logarithmic function dose-effect curve of tambulin on H520 cells for 48 h. The values were expressed as the means ± SD (n=6 for each group). *P <0.05 vs. 10 μg/ml; #P <0.05 vs. 20 μg/ml. [file Image_2.tif]

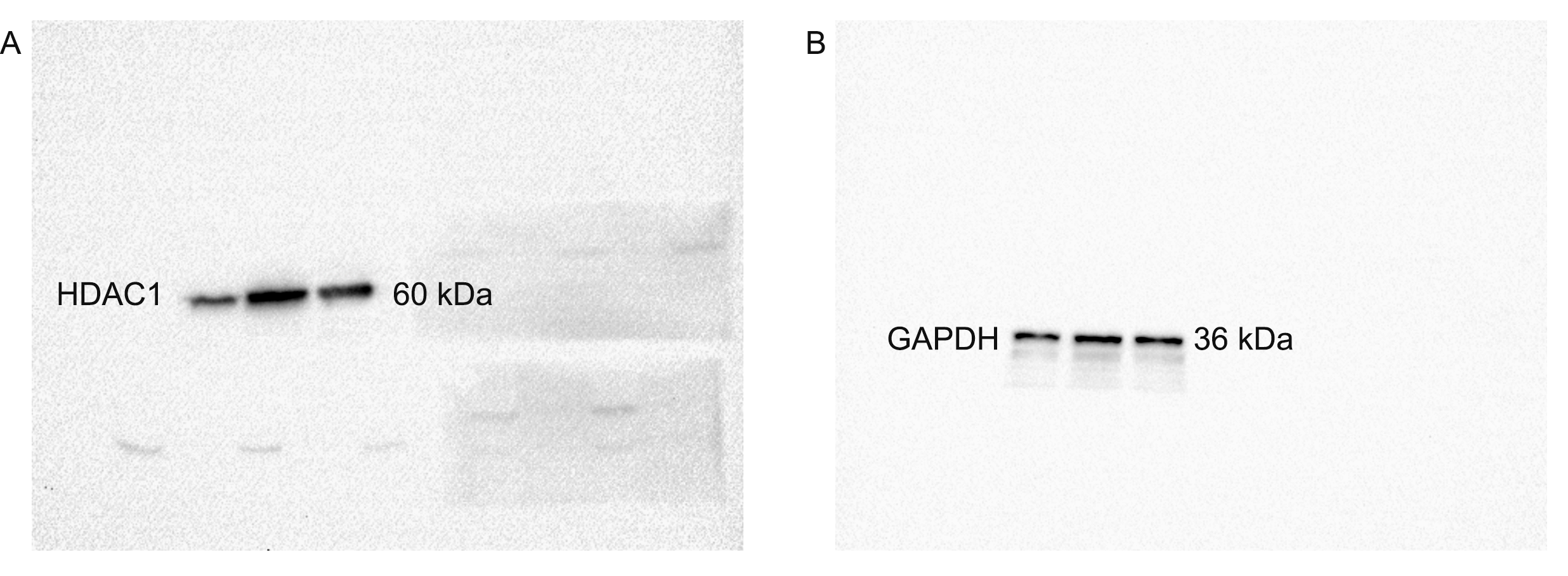

Supplement: Supplementary Figure 3 — The uncropped western blots of Figure 1. (A) The uncropped western blots of HDAC1. (B) The uncropped western blots of GAPDH. [file Image_3.tif]

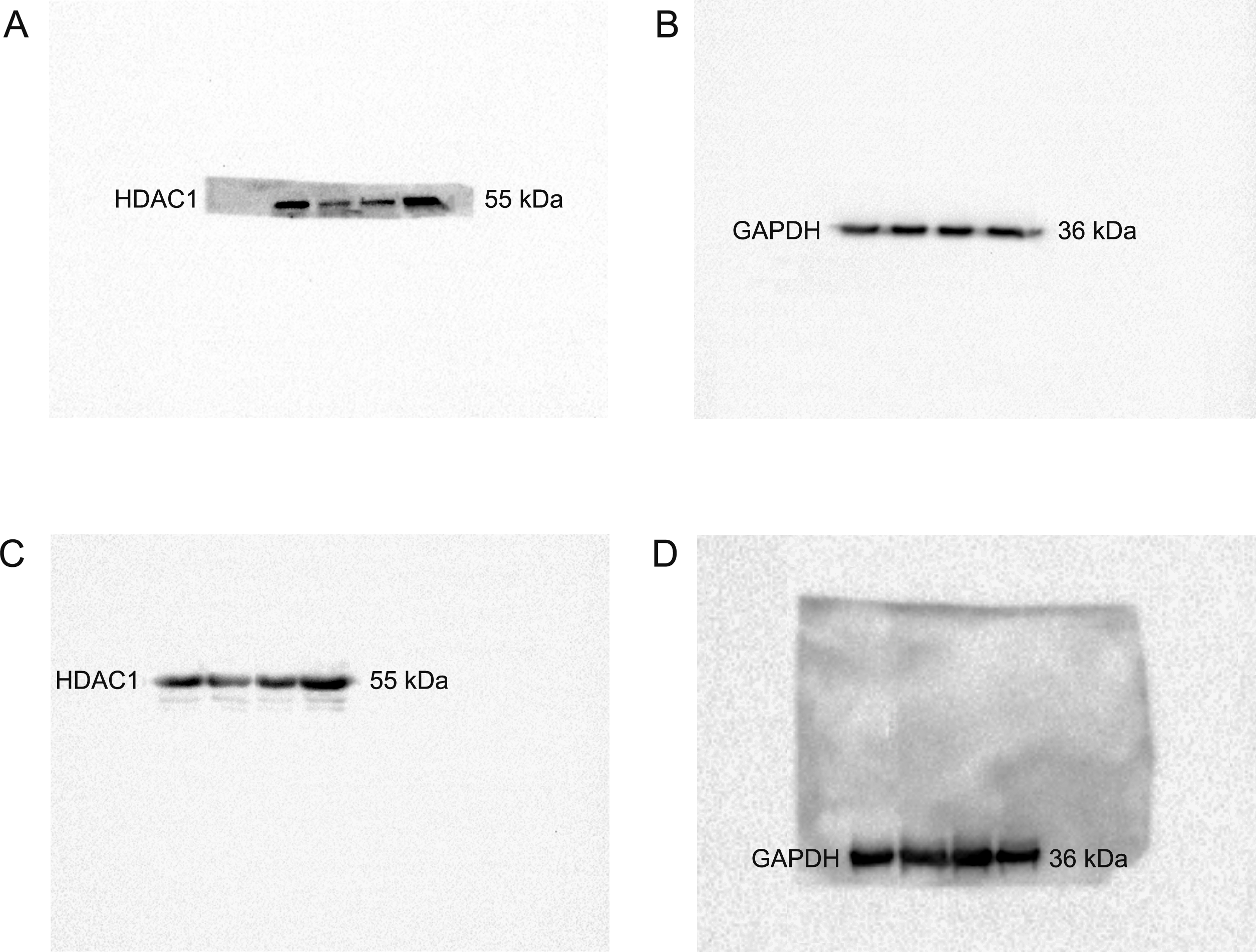

Supplement: Supplementary Figure 4 — The uncropped western blots of Figure 5. (A) The uncropped western blots of HDAC1 in H226. (B) The uncropped western blots of GAPDH in H226. (C) The uncropped western blots of HDAC1 in H520. (D) The uncropped western blots of GAPDH in H520. [file Image_4.tif]

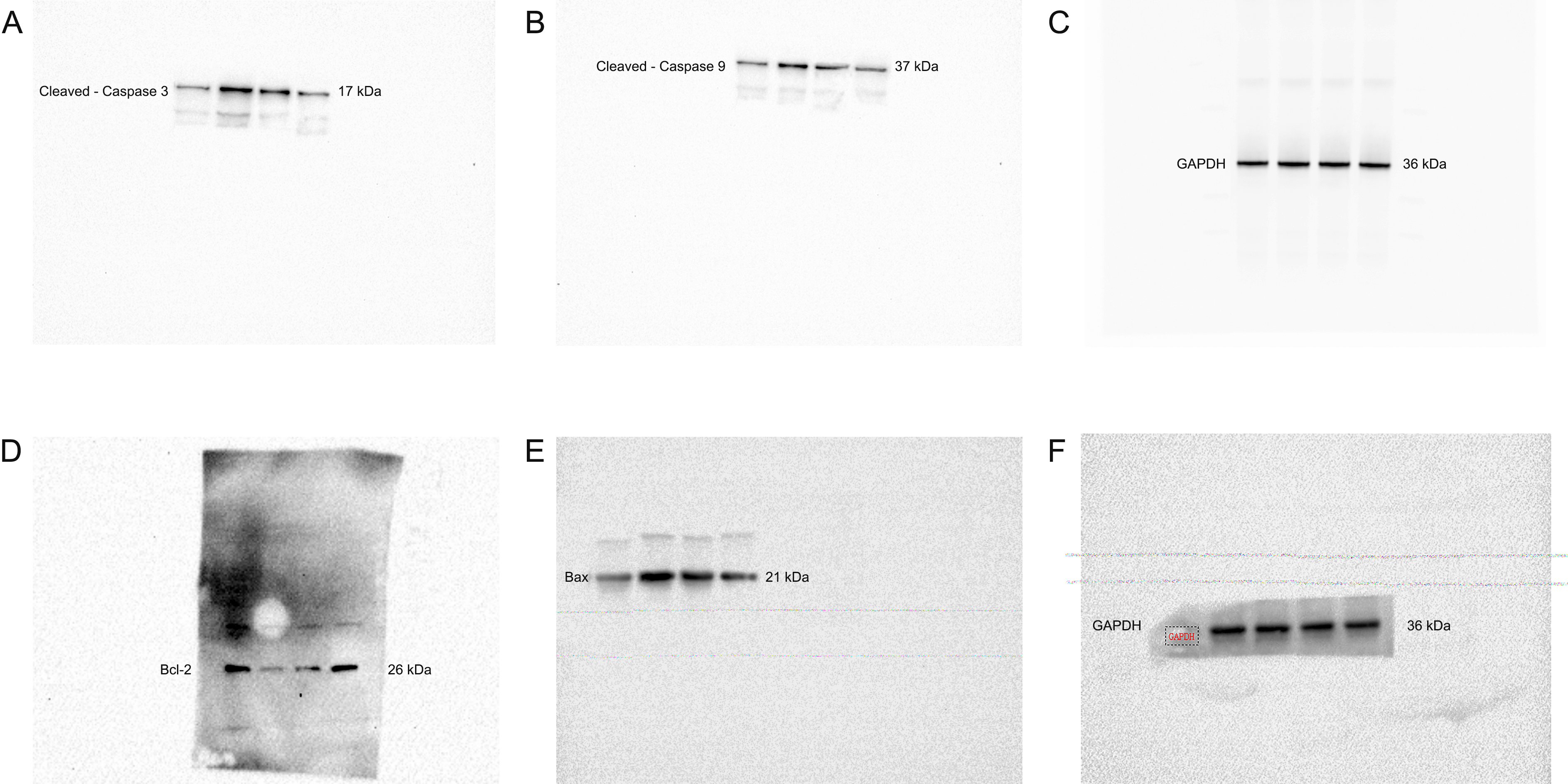

Supplement: Supplementary Figure 5 — The uncropped western blots of Figure 6. (A) The uncropped western blots of cleaved-caspase 3 in H226 cells. (B) The uncropped western blots of cleaved-caspase 9 in H226 cells. (C) The uncropped western blots of GAPDH in H226 cells. (D) The uncropped western blots of Bcl-2 in H226 cells. (E) The uncropped western blots of Bax in H226 cells. (F) The uncropped western blots of GAPDH in H226 cells. [file Image_5.tif]

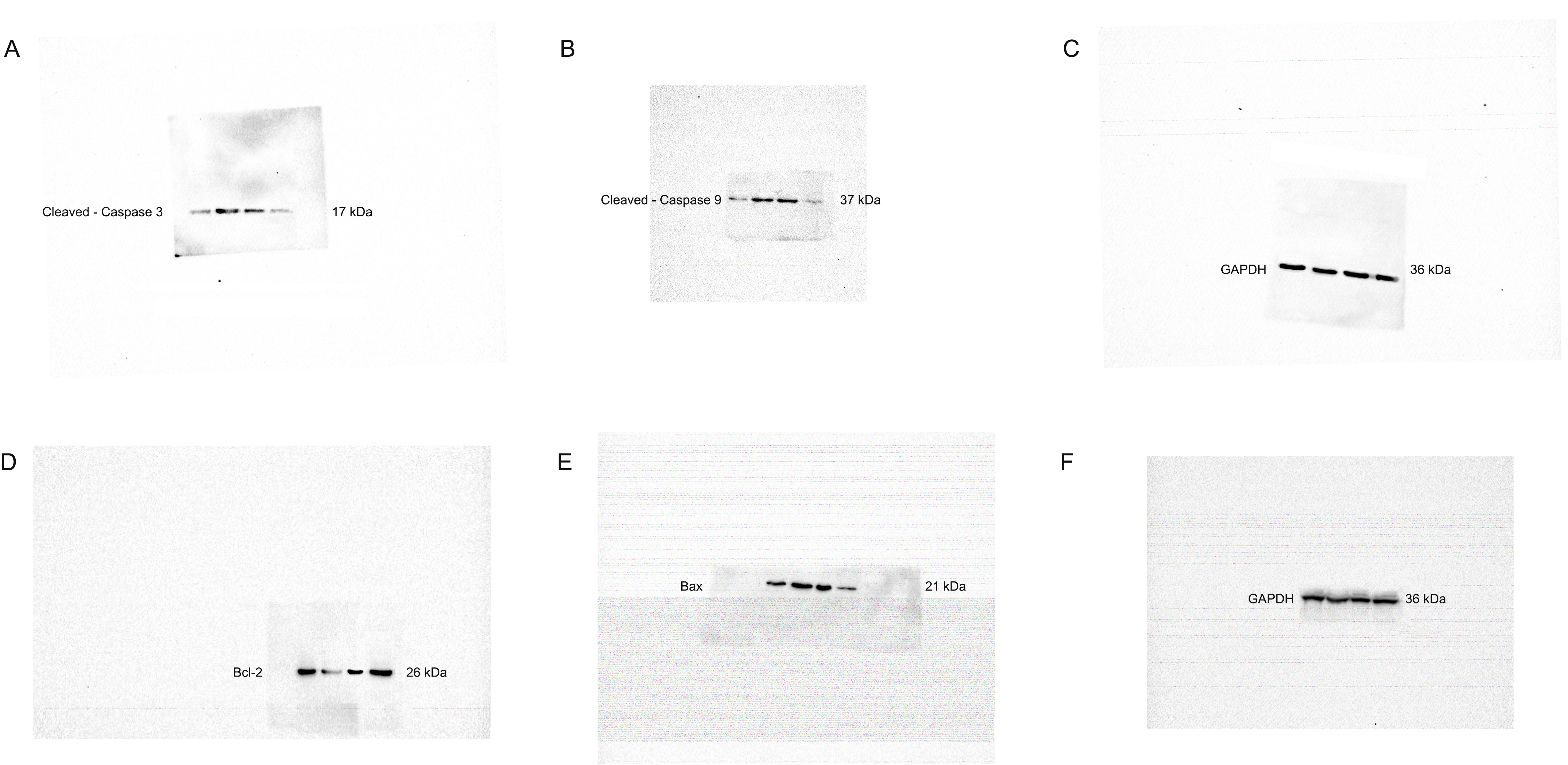

Supplement: Supplementary Figure 6 — The uncropped western blots of Figure 7. (A) The uncropped western blots of cleaved-caspase 3 in H520 cells. (B) The uncropped western blots of cleaved-caspase 9 in H520 cells. (C) The uncropped western blots of GAPDH in H520 cells. (D) The uncropped western blots of Bcl-2 in H520 cells. (E) The uncropped western blots of Bax in H520 cells. (F) The uncropped western blots of GAPDH in H520 cells. [file Image_6.tif]
